# Robustness of newt heads in condition of co-existence: a case of the Carpathian newt and the alpine newt

**DOI:** 10.1007/s00435-017-0366-7

**Published:** 2017-07-19

**Authors:** Mikołaj Kaczmarski, Anna Maria Kubicka, Martin Hromada, Piotr Tryjanowski

**Affiliations:** 1 Department of Zoology, Poznań University of Life Sciences, Wojska Polskiego 71C, 60-625 Poznań, Poland; 2 Laboratory and Museum of Evolutionary Ecology, Department of Ecology, University of Presov, 17 Novembra 1, 081 16 Prešov, Slovakia; 3 Department of Nature Conservation, Faculty of Biological Sciences, University of Zielona Gora, Zielona Gora, Poland

**Keywords:** Amphibians, Caudata, Geometric morphometrics, Museum collection, *Lissotriton montandoni*, *Ichthyosaura alpestris*

## Abstract

Co-existence between potentially competing newt species can lead to niche differentiation (e.g., in terms of diet shifts). This may cause adaptive responses involving changes in head shape. Here, we tested the hypothesis: the head shape of *Lissotriton montandoni* is different in conditions of co-occurrence with *Ichthyosaura alpestris* than in conditions in which other newt species are absent. We analysed images depicting head shape of specimens of *I. alpestris* and *L. montandoni* from a museum collection. All specimens of *I. alpestris* originated in a habitat where *L. montandoni* also occurred, whereas specimens of *L. montandoni* derived from populations that cohabited with *I. alpestris* and populations in which the presence of another newt species was not recorded. In each image, landmarks and semilandmarks were digitised. Females of *L. montandoni* from the population where *I. alpestris* also occurred were characterised by more massive heads and longer mouths in lateral views than females from sites where no other newt species occurred. Significant differences in head shape were also found when analysing ventral views between these species when they occupied the same habitat. We confirmed that the head shape of female *L. montandoni* differs between conditions of co-occurrence and absence of *I. alpestris*; no differences were found for males. A differently shaped head may be an adaptation to diet; *L. montandoni* females with longer mouths and more robust basal parts of the head can feed on larger invertebrates and compete more effectively with *I. alpestris*. The co-existence of newt species should be taken into account in future ecomorphological studies.

**Electronic supplementary material:**

The online version of this article (doi:10.1007/s00435-017-0366-7) contains supplementary material, which is available to authorized users.

## Introduction

Skull components in newts seem to be primarily related to foraging and feeding strategies (Rafiński and Pecio 1989; Malmgren and Thollesson 1999). Moreover, head shape may be strongly associated with different levels of aggression (Adams 2004) or with diet or interactions (Adams and Rohlf 2000). Some studies also indicate a high level of variation in head morphology due to sexual dimorphism in terms of size and shape in, for example, the genera *Lissotriton*, *Ichthyosaura*, and *Triturus* (Ivanović and Kalezić 2012). However, in spite of their close kinship and many synapomorphic features, to date patterns of sexual dimorphism in skull form between these species has not been determined (Ivanović and Kalezić 2012). In such studies, it is difficult to ascertain whether observed differences in head shape are due to sexual or ecological selection (Alcorn et al. 2013), especially given that head shape variation differs significantly between species and even between clutches within species, i.e., clutch effects (Adams 2011). Furthermore, the physical characteristics of the head are also impacted by environmental factors, and, at the same time, are highly correlated with the characteristics of the body as a whole (Adams et al. 2007). However, variability in body shape and size can also be a product of inter- and intraspecific interactions associated with occupancy of different niches in the presence of competitors (Adams and Rohlf 2000; Kniha et al. 2013). For example, the co-occurrence of *Lissotriton montandoni* (Boulenger, 1880) and *Ichthyosaura alpestris* (Laurenti, 1768) is associated with larger body sizes, but not differences in body shape in *L. montandoni* (Kniha et al. 2013). However, body size affects the anatomy of newts (Ivanović and Kalezić 2012), and therefore, a diverse habitat (in the context of the presence of other newt species) may influence the morphology of newts’ heads.

The co-existence of similar species occupying the same environment may lead to the formation of non-interactive and interactive communities (Vignoli et al. 2016). In the former, interactions between species are unimportant due to the availability of an unlimited number of niches (Sebastiano et al. 2012). However, in the latter, co-occurrence of species leads to competition, which is a strong and important driving force in the evolution of organisms (Connell 1978). Competition can regulate populations and influence community structure through, for example, mortality, growth rate, and fecundity, parameters, which ultimately determines the abundance of each species (Hixon and Johnson 2009). Limited food resources may lead to stronger competition within and between closely related species (Adams and Rohlf 2000); this, in turn, causes morphological, behavioural, or physiological differentiation, which does not appear when a habitat is free of competitors (Adams and Rohlf 2000). This means that some traits can be minimised or lost when the geographical ranges of species do not overlap (Brown and Wilson 1956; Dayan and Simberloff 2005). Changes in characteristics are important, because they reduce intra- and interspecific competition (Vignoli et al. 2007), and are known to occur in many taxa (Dayan and Simberloff 2005), including tailed amphibians (Adams and Rohlf 2000; Adams et al. 2007; Johanet et al. 2009; De Lisle and Rowe 2015). However, competition for food resources may also occur between sexes within the same species (De Lisle and Rowe 2015). Thus, competition can result in changes in sexual dimorphism, including the size and shape of the whole body or its various parts (e.g., the size of the head in most animal phyla, Shine 1989).

Modern European newt taxa (genera *Lissotriton, Ichthyosaura,* and *Triturus*) share many traits: they (1) occupy similar terrestrial and aquatic habitats; (2) exhibit similar life histories; and (3) are characterised by a narrow dispersal range and strong homing behaviour (Ivanović and Kalezić 2012). In *I. alpestris,* variation in the shape of the ventral cranium is more susceptible to adaptations to local environments than to phylogenetic constraints (Ivanović et al. 2009). The previous studies of habitat and spatial niche preferences found no evidence of partitioning of resources between even the most closely related newt species for example syntopic populations of the great crested newt *Triturus cristatus* (Laurenti, 1768) and the marbled newt *T. marmoratus* (Latreille, 1800) (Jehle et al. 2000). In addition, Kuzmin (1991) describes a high level of interspecific overlap between *I. alpestris* and *L. montandoni* during the larval phase: both species occur mainly at the base of, and within plants. However, during ontogeny, this relationship changes and syntopic post-metamorphs of both species exhibit a low degree of trophic overlap: during this phase, their diets are clearly different in terms of taxa and prey size (Kuzmin 1990). These two species are syntopic and frequently breed in the same water bodies even if their ecological requirements are not identical; this is especially true, because, in hilly or mountainous landscapes, the presence of suitable breeding zones is limited, causing isolation and restricting the distribution of newts (Świerad 1980; Plăiaşu et al. 2010).

Variability in skull shape in the Salamandridae family has been investigated in a few studies (e.g., Dandová et al. 1998; Malmgren and Thollesson 1999); however, most of the research focuses on head measurements, which are characterised by certain limitations. Recent advancement in geometric morphometrics enables the testing of more sophisticated biological hypotheses (Adams and Rohlf 2000; Ivanović et al. 2009; Adams 2011; Ivanović and Kalezić 2012), thanks to visualisation and statistical analysis of differences in the shapes of analysed objects.

Therefore, the main goal of this study is to analyse whether the presence of *I. alpestris* influences head shape in *L. montandoni*. The geographic range of both newt species overlap, and their biology and diet specialisation show similarities (Kuzmin 1990, 1991). Therefore, the co-occurrence of these two newt species may influence morphological traits (Kniha et al. 2013). Taking this into account, we tested the following hypothesis: the head shape of the Carpathian newt (*L. montandoni*) differs in conditions of co-occurrence with *I. alpestris* than in the absence of other newt species. To achieve our objective, we used geometric morphometrics, enabling comprehensive analysis of head shape. The results will determine whether the co-occurrence of newt species should be taken into account in future ecological studies. In addition, we would like to emphasise the value of museum collections in scientific research (Hromada et al. 2015).

## Materials and methods

The analysed material consisted of adult individuals of two newt species, *I. alpestris* (Laurenti, 1768) and *L. montandoni* (Boulenger, 1860) from the 75% alcohol-preserved herpetological collections from the Šarišské múzeum (Šariš Museum, Bardejov, Slovakia) (Hromada et al. 2015; Kaczmarski and Baranová 2015). All specimens were collected in the Bardejov region between 1958 and 1976 (for more details, see Table 1 and Kaczmarski and Baranová 2015).

**Table 1:** List of sites included in the study

Type of site	Site	Date		LM		IA	
				Female	Male	Female	Male
LM and IA	Kurov Kurovskie sedlo	12-30.IV.1976		40	46	28	33
			Mean	40	46	28	33
LM	Krize pod dedina	26.V.1958		36	37		
	Bardejov urbamovka	1.V.1959		8	30		
			Mean	44	67		

LM, *Lissotriton montandoni*; IA, *Ichthyosaura alpestris*

In Slovakia (Carpathian region), four newt species occur (Baruš and Oliva 1992). Small, brown, smooth newts, *L. vulgaris* (Linnaeus, 1758) and *L. montandoni*, occur sympatrically with the larger *I. alpestris* (a medium-sized newt) and *Triturus cristatus* (a large newt; the morphologically classified by size according to Zajc and Arntzen 1999). Both studied species, *I. alpestris* and *L. montandoni*, are predators and occupy a similar terrestrial niche (Janiga and Mlichová 2004; Kniha et al. 2013; Sparreboom 2014). Moreover, both rely on small water reservoirs, marshes, puddles, or small pools and wheel ruts (Szymura 1974; Kuzmin 1990; Babik and Rafinski 2001; Kniha et al. 2013), which are highly vulnerable to degradation. On a wider scale, European newts (previously classified within the traditional genus *Triturus*) are vulnerable to severe decline, as they are affected by the expansion of agriculture, wetland pollution, and the introduction of fish species (Denoël 2012). They are also gregarious throughout their lives and tend to live in high-density aggregations during the breeding season and juvenile stage (Sparreboom 2014).

All *I. alpestris* individuals (female and male) derive from similar habitats where *L. montandoni* also occurs (syntopic sites). We did not analyse the head shape of *I. alpestris* from environments where the presence of another newt species was not recorded, because in this region of Slovakia, *I. alpestris* always co-exists with other newt species (for details, see Kaczmarski and Baranová 2015). Specimens of *L. montandoni* derive from two different populations, sympatric, and allopatric. The former group occupied the habitat where *I. alpestris* occurred, while the presence of another newt species was not recorded in the habitat of the latter group.

### Digitising landmarks and semilandmarks

We photographed ventral and right-lateral views of the head with a scale for each specimen using a Pentax Optio WG-5 GPS digital camera in Digital Microscope Mode, with an LED ring flash and macrostand. The ventral surface of the head was placed upon the glass as flat as possible, whereas the lateral part of the head was placed perpendicular to the glass. The camera was mounted on the glass in such a way that all photographs were taken at the same distance from the head and the head was always in the same position. This methodology had been created and tested for a previous study (see Kaczmarski et al. 2015).

**Fig. 1:**
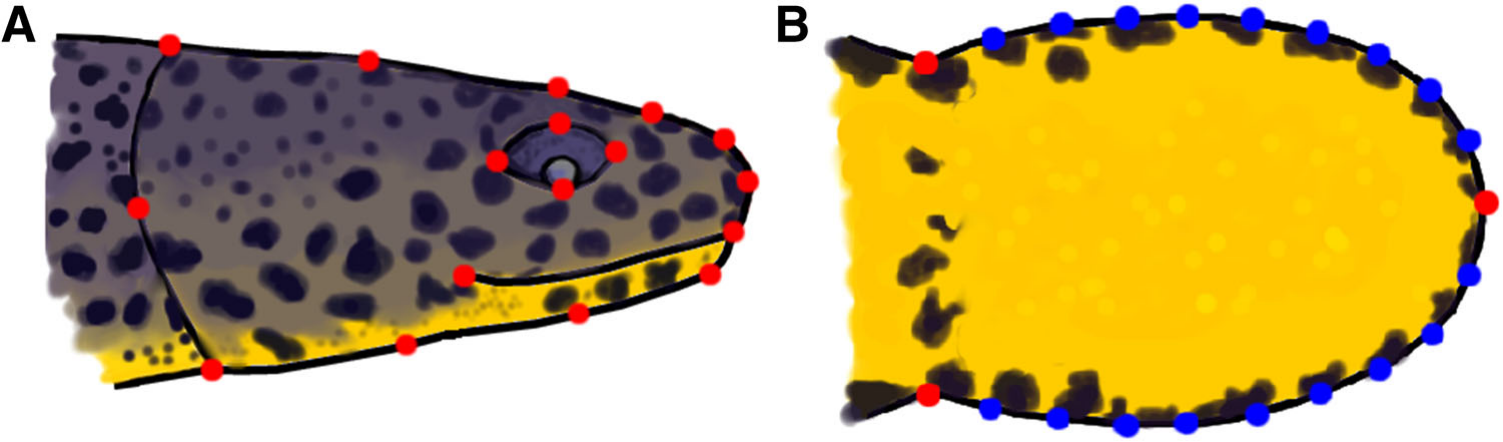
**a** Alpine newt (IA) head with digitised landmarks (*red dots*) in the lateral view; **b** a newt head with digitised landmarks (*red dots*) and semilandmarks (*blue dots*) in the ventral view

Subsequently, 17 landmarks were digitised in the same direction on every image of the right-lateral view of the head (Fig. 1a). On every image of the ventral view, 3 landmarks and 18 semilandmarks were digitised (Fig. 1b). This part of the research was carried out using tpsDig2 software (Rohlf 2010). The ventral view of the newt head has very few homologous points; we, therefore, used semilandmarks to analyse shape differences within and between species.

### Geometric morphometric analysis

To estimate measurement error, two photographs were taken of each newt head in dorsal and lateral views for 15 individuals. Next, on the extra set of photographs, landmarks and semilandmarks were digitised twice by one observer. Analysis of variance (Procrustes ANOVA) was used to quantify measurement error at two levels (imaging and digitising) separately for the ventral and lateral views (all *p* < 0.05; Klingenberg et al. 2002).

Prior to statistical analysis, all landmark and semilandmark configurations were superimposed using a generalised Procrustes analysis (GPA). This method is used to compare shapes through translation, rotation, and standardisation of each object to unit centroid size, which is a measure of scale and is not associated with the shape in the absence of allometry (Zelditch et al. 2012). Centroid size (CS) was calculated as the square root of the summed squared distances of each landmark from the centroid of the landmark configuration of the newt head in the lateral and ventral view (Mitteroecker and Gunz 2009; Alarcón-Ríos et al. 2017).

Then, Procrustes MANOVA test was used to analyse whether the lateral and ventral views of the head shape in *L. montandoni* differed significantly between the habitats with co-occurrence, and habitats in which other newt species were absent (*Kríže pod dedina* and *Bardejov urbanovka*, Table 1). Following Procrustes MANOVA, multivariate regression of shape variables on size (CS) was carried out to characterise the allometry of newt heads. To test the significance of these associations, a permutation test for pairwise distances of 10,000 randomised rounds was performed (with a significant acceptance level at *p* < 0.05).

Next, a principal component analysis (PCA) and a canonical variate analysis (CVA) were performed. The PCA was used to simplify descriptions of variation among newt populations within species, sexes, habitat type, and sites. A canonical variate analysis (CVA) was used to analyse and simplify descriptions of differences between two habitat types (where interspecific competition occurred or was not recorded) within species and sexes. To test the significance of these shape differences, a permutation test for pairwise distances of 10,000 randomised rounds of CVA was carried out. The significance level of the CVA was Bonferroni-adjusted (Sokal and Rohlf 1995) to 0.05/15 = 0.0033 owing to the large number (15) of comparisons. All geometric morphometric analyses were carried out using MorphoJ (Klingenberg 2011).

### Centroid size analysis

Centroid size was used to analyse differences in the head size within species, sex, habitat type of *L. montandoni,* and sites. For this purpose, we calculated the centroid size for each individual in two views (ventral and lateral) and using a traditional statistical approach to analyse the data. Descriptive statistical analyses such as mean and standard deviation of the centroid size in the both views (ventral and lateral) were calculated for each population within species and sex. Shapiro–Wilks test showed the normality of ventral (*W* = 0.98673, *p* > 0.05) and lateral centroid size (*W* = 0.9919, *p* > 0.05). A generalised linear model (GLM) was used to test whether the centroid size in the both view was associated with factors such as species newt, sex, habitat type or site. Four models were created, which were analysed using the package “MuMIn” (Bartoń 2016) to estimate the best fitting model. The centroid size was the dependent variable and species, sex, habitat type, and site were covariables. The GLM was calculated for the ventral and lateral view separately. All statistical analyses were carried out using R software (version 0.00.902).

## Results

The mean squares for the two levels of error (imaging and digitising) were smaller than for an individual in the lateral view (Table 2). Similar results were obtained in the ventral view: all types of error were characterised by smaller values of mean squares than individual and side-by-individual interaction (Table 2). This means that the measurement error was smaller than the smallest level of biological variation. We can, therefore, conclude that imaging or digitising error did not affect the obtained results.

**Table 2:** Results of the Procrustes ANOVA for two levels of error in a study of the newt heads

Effect	Sum of squares	Mean squares	*df*	*F*	*p*	Pillay’s trace	*p*
Lateral view							
Individual	0.35606337	0.0008477699	420	28.55	<0.0001		
Imaging	0.01336254	0.0000029694	450	1.00	0.5007	9.93	0.5738
Digitising	0.02674891	0.0000029772	900				
Ventral view							
Individual	0.13243112	0.0004978614	266	10.11	<0.0001		
Side	0.00019939	0.0000104940	19	0.21	0.9999		
Individual × side	0.01309395	0.0000492254	266	10.55	<0.0001	16.57	<0.0001
Imaging	0.00265844	0.0000046639	570	1.36	<0.0001	6.14	0.6852
Digitising	0.00392042	0.0000034390	1140				

**Fig. 2:**
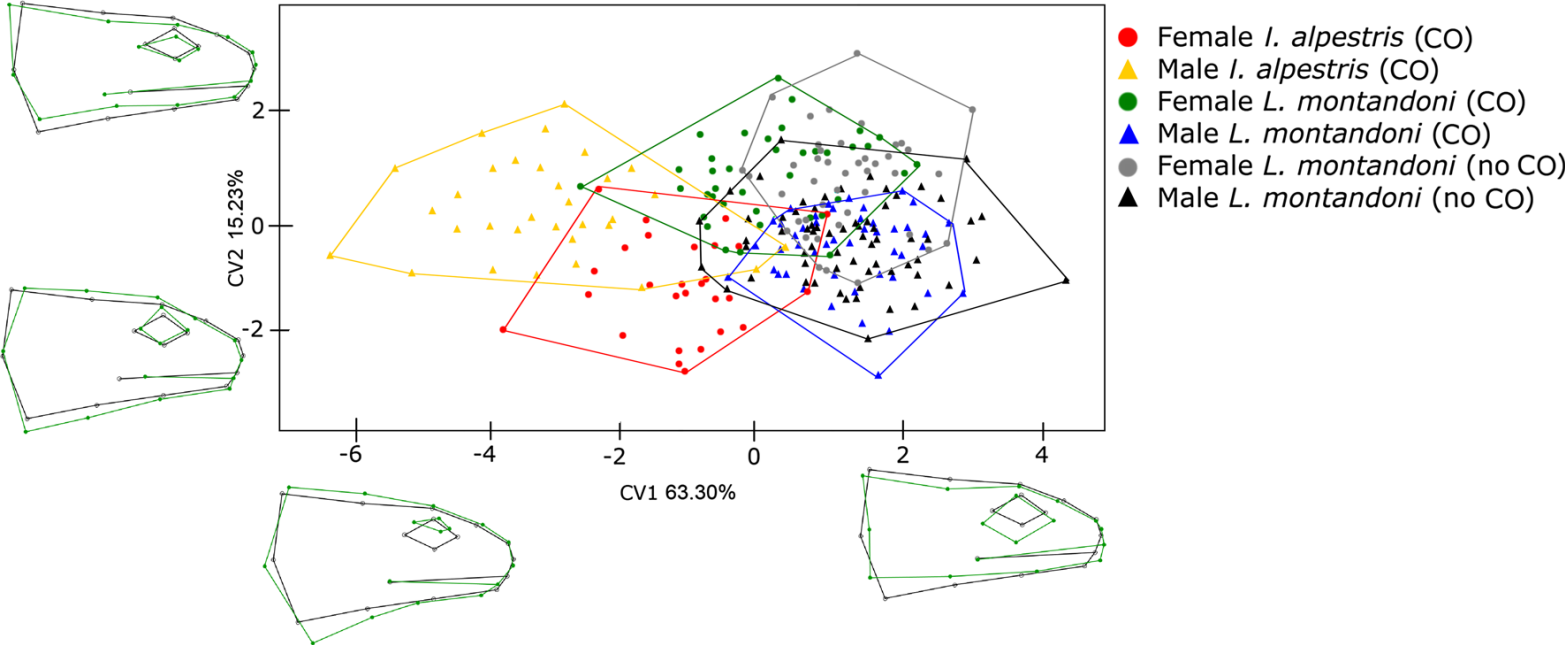
CVA of shapes of newt heads (*lateral view*). *CO* co-occurrence with other newt species, *no CO* absence of other newt species, *black lines* mean shape, *green lines* shape representing a specific CV

The Procrustes MANOVA test revealed no significant differences in the lateral and ventral views of head shape in *L. montandoni* between two habitats in which co-occurrence of another newt species was not recorded (see, Table 1S). Allometry was revealed in the analysed material (ventral view: predicted = 10.22%, *p* = 0.0001; lateral view: predicted = 5.56%, *p* = 0.001). To ensure the allometric component and size-related differences between the groups had been removed, PCA and CVA were carried out using the residual components. The graphical results of the PCA for the newt species within sex, habitat type, and site are shown in Figs. 1S and 2S. PC1 explains 28.51% of variation in the material, while PC2 is responsible only for 17.96% of variation in the lateral view of the head shape. PC1 describes variation in the head high and the head length, while PC2 explains variation in the mouth length. However, all PCA-created groups overlap, with females *I. alpestris* characterised by the greatest variation in the lateral view. PC1 explains 78.43% of variation in the material, while PC2 is responsible only for 11.19% of variation in the ventral view of the head shape. PC1 describes variation in the head width (near the shoulder) and the head length, while PC1 is responsible for the head slimness. Again, all PCA-created groups overlap, with females and males *I. alpestris* characterised by the greatest variation in the ventral view.

The results of the permutation test in CVA between the two populations within sex and species are shown in Tables 3 and 4. The CVA shows that all differences in the lateral view of head shape are significant (*p* < 0.0033; Table 3) except differences between sexes (in *I. alpestris* and *L. montandoni*) and *L. montandoni* males from habitats where *I. alpestris* occurs and does not occur (*p* < 0.0033; Table 3). CV1 explains 63.30% of variation in the analysed material, while CV2 is responsible for 15.23% of variation in the lateral view of head shape. CV1 and CV2 separated each group from the others, although some lateral views of head shape in *I. alpestris* and *L. montandoni* overlap. Individuals with positive values of CV1 are characterised by bigger eyes, higher located mouths and slimmer heads, than those with negative values of CV1. CV2 explains the concavity and convexity of the middle part of the head (Fig. 2). *L. montandoni* females from the site where *I. alpestris* occurs are characterised by higher, and more massive heads in the part near the shoulders and the mouth is located lower on the face than females from the second habitat (without *I. alpestris*). In *L. montandoni* from both habitats (with and without *I. alpestris*), males are characterised higher head-convexity heads and a shorter mouth than females of the same species.

**Table 3:** Results of the permutation test for pairwise distances in CVA for lateral views of head shape

	F IA (CO)	M IA (CO)	F LM (CO)	M LM (CO)	F LM (no CO)
M IA (CO)	0.0236				
F LM (CO)	**0.0001**	**0.0001**			
M LM (CO)	**0.0001**	**0.0001**	0.0104		
F LM (no CO)	**0.0001**	**0.0001**	**0.0003**	**0.0023**	
M LM (no CO)	**0.0001**	**0.0001**	**0.0001**	0.0702	0.0345

Bold indicates significant differences between shapes at *p* < 0.0033 (after a Bonferroni correction)

*M* male, *F* female, *IA I. alpestris*, *LM L. montandoni*, *CO* co-occurrence with other newt specie, *no CO* lack of other newt specie

**Table 4:** Results of the permutation test for pairwise distances in CVA for ventral views of head shape

	F IA (CO)	M IA (CO)	F LM (CO)	M LM (CO)	F LM (no CO)
M IA (CO)	0.3416				
F LM (CO)	0.0117	**0.0001**			
M LM (CO)	0.0964	**0.0009**	0.0984		
F LM (no CO)	0.0543	**0.0003**	0.2436	0.3790	
M LM (no CO)	0.1494	**0.0013**	0.0079	0.3990	0.0996

Bold indicates significant differences between shapes at p < 0.0033 (after a Bonferroni correction)

*M* male, *F* female, *IA I. alpestris*, *LM L. montandoni*, *CO* co-occurrence with other newt specie, *no CO* lack of other newt specie

**Fig. 3:**
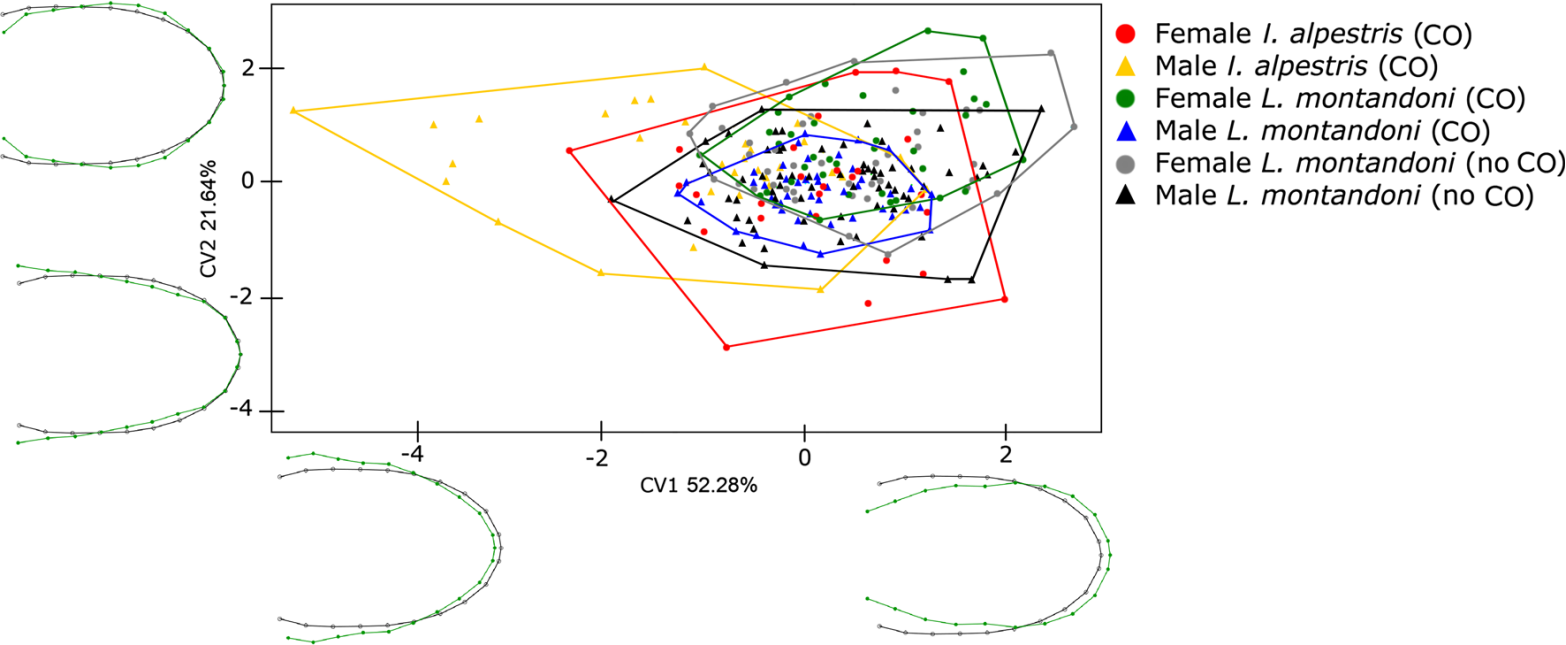
CVA of shapes of newt heads (*ventral view*). *CO* co-occurrence with other newt species, *no CO* absence of other newt species, *black lines* mean shape, *green lines* shape representing a specific CV

CVA shows that not all heads differ significantly in the ventral view. A significant sexual dimorphism was not revealed (*p* < 0.0033; Table 4). Moreover, *I. alpestris* males are characterised by a significantly different ventral head shape compared to *L. montandoni* females from the habitat without *I. alpestris* and *L. montandoni* males from both habitats (with and without *I. alpestris*; Table 4). Figure 3 shows that CV1 is responsible for 52.38%, while CV2 explains 21.64% of variation in the ventral view of the head shape. CV1 describes variation and maximal differences in the length and width of the head from the middle part to the base. CV2 explains only variability in the width of the part the head nearest to the shoulders. Individuals with positive values of CV1 are characterised by longer, but narrower, heads in the ventral view than those with negative values of CV1, whereas individuals with extreme positive values of CV2 are characterised by wider heads near the apex and slightly narrower heads near the shoulders than those with negative values of CV2.

Descriptive statistics of the centroid size are tabulated in the supplement (Table 2S). In the ventral view, females from all populations were characterised by greater values of centroid size. In turn, in the lateral view, males generally exhibited greater values of centroid size than females. The GLM model, with the covariables such as species newt, sex, habitat type, and site, was estimated as the best fitting model in the ventral and lateral views. All covariables in the model for the both view were significant (*p* < 0.05, see details, Table 5).

**Table 5:** Results of GLM multivariate analysis with centroid size as a dependent variable

	Estimated std.	Error	*t* value	*p*
Ventral view				
Intercept	22.5674	0.2386	94.570	<2e−16***
Species	−2.2672	0.1786	−12.697	<2e−16***
Sex	−1.0698	0.1411	−7.580	5.77e−13***
Habitat	1.0391	0.3484	2.982	0.00313**
Site	−0.5622	0.2302	−2.443	0.01523*
Lateral view				
Intercept	15.98305	0.26791	59.658	<2e−16***
Species	−1.49592	0.17681	−8.461	1.8e−15***
Sex	−1.23465	0.10754	−11.481	<2e−16***
Habitat	0.46481	0.12012	3.870	0.000137***
Site	−0.35276	0.09908	−3.561	0.000438***

* 0.01, ** 0.001, *** 0.0001

## Discussion

This study shows that *L. montandoni* is characterised by different head shape, depending on whether *I. alpestris* occurs or not in a habitat; however, this variation is found only in lateral views of females. Our results contrast with those of a study by Kniha et al. (2013) who stated that co-occurrence of *I. alpestris* affects the body size, but not the shape of *L. montandoni*. This discrepancy may be caused by the use of different methodologies. Kniha et al. (2013) only used four characteristics (body length, tail length, head width, and weight), which do not enable detailed interpretation of changes in body and head shape. Another cause of this discrepancy might be a lack of separate analyses by sex. Sexual dimorphism may be important, since our GM analysis shows that occupancy of a habitat by both *L. montandoni* and *I. alpestris* influences only *L. montandoni* females.

The differences in both newt species were also related with the head size, which was described in our study as a centroid size. The *L. montandoni* individuals from the habitats where *I. alpestris* was also noted were characterised by smaller heads (in both views). The smaller dimensions may be the result of adaptation to local conditions where *I. alpestris* co-occurs and availability of food is limited (Ivanović et al. 2009; Ivanović and Kalezić 2012; De Lisle and Rowe 2015). The GLM results show that many factors, such as newt species, sex, habitat type, and site, influence head size. This can suggest that the variation in head size is more prone to environmental factors and processes than to phylogenetic constrains (Ivanović et al. 2009). Moreover, the *I. alpestris* and *L. montandoni* females were characterised by larger heads than males, but only in the ventral view. This may be a result of differences in ecological demands due to diet specialisation, especially that the ventral cranium is associated with foraging and feeding (Ivanović and Kalezić 2012).

The major anatomical difference between *L. montandoni* females from the two analysed habitats (1: *I. alpestris* and *L. montandoni*; 2: *L. montandoni*) (i.e., the variation in the robustness and length of the mouth) can be interpreted biomechanically. *L. montandoni* females from the habitat where *I. alpestris* was recorded are characterised by higher and larger heads in the part near the shoulders and mouths with locations lower than those of females from the site where only *L. montandoni* occurs. The observed changes in head shape found in *L. montandoni* females can be associated with prey consumption, since prey size affects feeding mechanics and correlates with body size in amphibians (Larsen and Guthrie 1975; Reilly and Lauder 1989; Zerba and Collins 1992; Werner et al. 1995; Denoël and Andreone 2003).

Another factor influencing head shape in newts is the co-existence with closely related species characterised by a similar body size and food niche; this can have a number of consequences manifested by changes in adaptations to the environment, behaviour, or morphological traits (Adams 2011). Tailed amphibian species exhibit variations in head shape associated with food resources (Adams and Rohlf 2000), behavioural aggression (Adams 2004, 2010), and genetic covariance (Adams 2011). Since the similarity between *L. montandoni* and *I. alpestris* is great, the observed differences in head shape in *L. montandoni* females may be a consequence of the co-occurrence of these two newt species (*L. montandoni* and *I. alpestris*).

Head shape patterns are present at earlier ontogenetic stages, which means that differences in head shape between species and between clutches may occur in the early development (Adams 2011). However, the observed variability may also be closely related to the density and availability of food (De Lisle and Rowe 2015) or habitat differences (Sotiropoulos et al. 2008; Johanet et al. 2009), which can strongly influence morphological traits. The diets of newt larvae belonging to medium-sized (*I. alpestris*) and small (*L. montandoni*) species are more similar than those of larvae belonging to *L. montandoni* and *Triturus cristatus.* Furthermore, this degree of overlap in the ecological niches of *L. montandoni* and *I. alpestris* remains high until metamorphosis (Kuzmin, 1991). However, analysis of diets suggests different food resource allocation among larval newts rather than food competition during the early stages of *L. montandoni* and *I. alpestris* development (Kuzmin 1991).

*Lissotriton montandoni* and *I. alpestris* adults exhibit a lower degree of trophic overlap than their larvae due to different patterns of foraging activity. This is because the Urodela guild comprises generalist (in terms of feeding behaviour) species during the aquatic phase, and the habitat can be partitioned in three dimensions (with different microhabitats within the water column) (Vignoli et al. 2016). Adult individuals of *I. alpestris* prefer terrestrial prey, whereas *L. montandoni* adults choose a more aquatic diet (Kuzmin 1990), although sexual dimorphism was not taken into account in this study, and the results should, therefore, be interpreted with caution.

Migration phenology may also account for these differences. Lack of significant differences in the head shape of *L. montandoni* males from the two habitats may be due to their arrival in breeding areas before the females (Semlitsch et al. 1993). *Lissotriton montandoni* females arrive at a pond at a time when smaller *L. montandoni* males and similarly sized *I. alpestris* males are already established in an aquatic environment; *L. montandoni* females also start to use resources at the same time with *I. alpestris* females. In the case of habitats lacking trophic resources, or characterised by the presence of a second species (in this case *I. alpestris*), individuals may shift their foraging to a terrestrial environment (Covaciu-Marcov et al. 2010). This switch might be easier adaptation for females, because they have no toe webbing and are characterised by a more massive shoulder girdle and more powerful jaws. Therefore, in our opinion, the stronger pressure of selection to optimise feeding in *L. montandoni* females, compared with males, may be the reason for the observed differences between the habitats in the head shape of females only.

The head shape did not show any patterns of sexual dimorphism in the newt species. On the other hand, the GLM test showed that females of *L. montandoni* and *I. alpestris* are characterised by significantly greater head size (measured as CS) than males. The results are consistent with a study by Ivanović and Kalezić (2012) who noted sexual dimorphism in size, but not in shape, in the head *I. alpestris*. Other researchers have also shown significant differences in head size between sexes in *L. montandoni* (Dandová et al. 1998; Janiga and Mlichová 2004). Furthermore, it has been found that the stomach of *L. montandoni* males is more frequently empty, when compared to females, which might be associated with males’ higher motivation for mating than in feeding (Covaciu-Marcov et al. 2010). Moreover, the size and nutrition of the female is closely linked to fertility (Hayes et al. 2010). As a result, females exhibit more intense feeding than males during the mating season. This may explain why *L. montandoni* and *I. alpestris* females are characterised by greater head dimensions. The lack of sexual shape dimorphism in our study may suggest that male and female *L. montandoni* and *I. alpestris* have similar feeding strategies (Malmgren and Thollesson 1999).

*Ichthyosaura alpestris*’*s* adaptation to a terrestrial environment can be seen in its head shape, which is more massive near the shoulders, and the lower location of its mouth, compared to *L. montandoni* individuals. This is consistent with known changes in prey capture mechanics of *I. alpestris* over the seasons due to increased feeding success in both environments (aquatic and terrestrial) through elaboration of a lingual prehension mechanism in the terrestrial phase (Heiss et al. 2013).

Four species of newts can occur in syntopy in the Western Carpathian region, but at higher altitudes, only *I. alpestris* and *L. montandoni* are present (Babik and Rafinski 2001). Occurring in the same area, these newts are described as a guild; they share major ecological requirements, such as breeding ponds or other stagnant-water habitats (Kuzmin 1991; Van Buskirk 2007; Denoël and Ficetola 2008; Denoël 2012). If, as according to Van Buskirk (2007), the interaction coefficient is somewhat greater among species pairs with large size differences, we suppose that the effect of the presence of *I. alpestris* on small-bodied newts (*Lissotriton*) is similar to that of large-bodied newts (*Triturus* complex) on *I. alpestris* (Van Buskirk 2007). Therefore, the methodology of morphological research should take into account potential interspecific influences and species guilds. Furthermore, with regard to a previous work on alpine newts (*I. alpestris*) on the patterns of skull size and shape variation to molecular phylogeny (Ivanović et al. 2009), we would like to disregard Ivanovic’s analysis in the context of the occurrence of different species at each site and the character of the local newt guild.

In a previous study, the authors showed that habitat does not influence size or shape in *L. montandoni* (Kniha et al. 2013). However, exclusive occupancy of a habitat by small newts, such as *L. montandoni,* may suggest that this species is more flexible and capable of occupying habitats, whereas medium-sized newts (e.g., *I. alpestris*) are characterised by a shortage of food or lack of space (Kniha et al. 2013). No size differences between *L. montandoni* from different altitudes in the Tatra Mountains have been recorded (Janiga and Mlichová 2004), although differences have been detected between the Tatras (high mountains) and Poloniny National Park (lower mountains) (Kniha et al. 2013). This confirms that most amphibians generally reach a greater body size at higher latitudes, and in cooler environments (Ashton 2002). However, our samples were collected from sites located at a short distance from each other; thus, latitude is not probably responsible for differences in head size (CS) among the *L. montandoni* populations, but differences in environments.

In this study, we used geometric morphometrics to identify and describe differences in the head shapes of two newt species. Using this method, we were able to link important anatomical changes in head shape to the co-occurrence of two related newt species. However, the data presented here only reflect a snapshot in time, so the recorded differences in head shape of *L. montandoni* and *I. alpestris* may be of a temporary nature and thus may require further study conducted on other populations. All analysed specimens were captured in April–May, while this period falls within the peak of reproductive aggregation of adults in the pond (Baruš and Oliva 1992). The beginning and dynamics of the breeding season are determined mainly by water and air temperature. Adults ready for reproduction arrive at the pond and, as the temperature rises, their mating behaviour increases (Sparreboom 2014). Furthermore, at the same time, tail fins in both of the sexes become flatter, which makes it easier to swim, as well as perform mating dances. All the specimens were captured at the mating peak when both sexes were in abundance in the water (Stanislav Trenčan—retired taxidermist in SMB, personal comments). The season of capture closely reflected the population variability between the year and local conditions. This is why we believe that the collectors’ method of sampling has allowed us to gather representative samples for both of the sites as well as the population as a whole, whilst the samples reflect well the variability of the local populations.

However, we believe that the demonstrated plasticity in head shape may result from the presence of another species. To test this, morphological differentiation in head shape in newts could be analysed in the context of ecological character displacement, which occurs to reduce competition between two species. Unfortunately, the museum collection used in this study did not fulfil a set of criteria which must be satisfied to test the hypothesis of character displacement (Schluter and McPhail 1992; Dayan and Simberloff 2005). Further studies on living adult newts in the context of character displacement should, therefore, be carried out.

Museum specimens offer an opportunity to preform research without harvesting or killing animals (Hromada et al. 2015; Talley et al. 2015). Thus, inventory and digitisation of collections are an important step forward. Whenever possible, specimens should be preserved over the long term (Zimkus and Ford 2014), which requires scientific curation and appropriate funding (Kress 2014). Unfortunately, in contrast to mammals or birds, amphibians are often kept in poor conditions (e.g., collectively in jars or tanks) (Hromada et al. 2003; Kaczmarski and Baranová 2015). In an era of global extinctions of amphibians (Stuart et al. 2005), collections, such as the one from the Bardejov Sarriske Museum, should be properly secured and maintained for future study.

## Electronic supplementary material

Below is the link to the electronic supplementary material.

Fig. 2S PCA of shapes of newt heads (ventral view). CO, co-occurrence with other newt species; no CO, absence of other newt species; black lines, mean shape; green lines, shape representing a specific CV; KK, a population from Kurov Kurovskie sedlo; KD, a population from Krize pod dedina, BU, a population from Bardejov urbamovka (TIFF 400 kb)

Fig. 1S PCA of shapes of newt heads (lateral view). CO, co-occurrence with other newt species; no CO, absence of other newt species; black lines, mean shape; green lines, shape representing a specific CV; KK, a population from Kurov Kurovskie sedlo; KD, a population from Krize pod dedina; BU, a population from Bardejov urbamovka (TIFF 509 kb)

Supplementary material 3 (DOCX 13 kb)

Supplementary material 4 (DOCX 13 kb)
