# Supplementary figures and images for: Robustness of newt heads in condition of co-existence: a case of the Carpathian newt and the alpine newt
Source: Zoomorphology. 2017 Jul 19;136(4):511–21. doi: 10.1007/s00435-017-0366-7 (PMC5653732; doi:10.1007/s00435-017-0366-7)

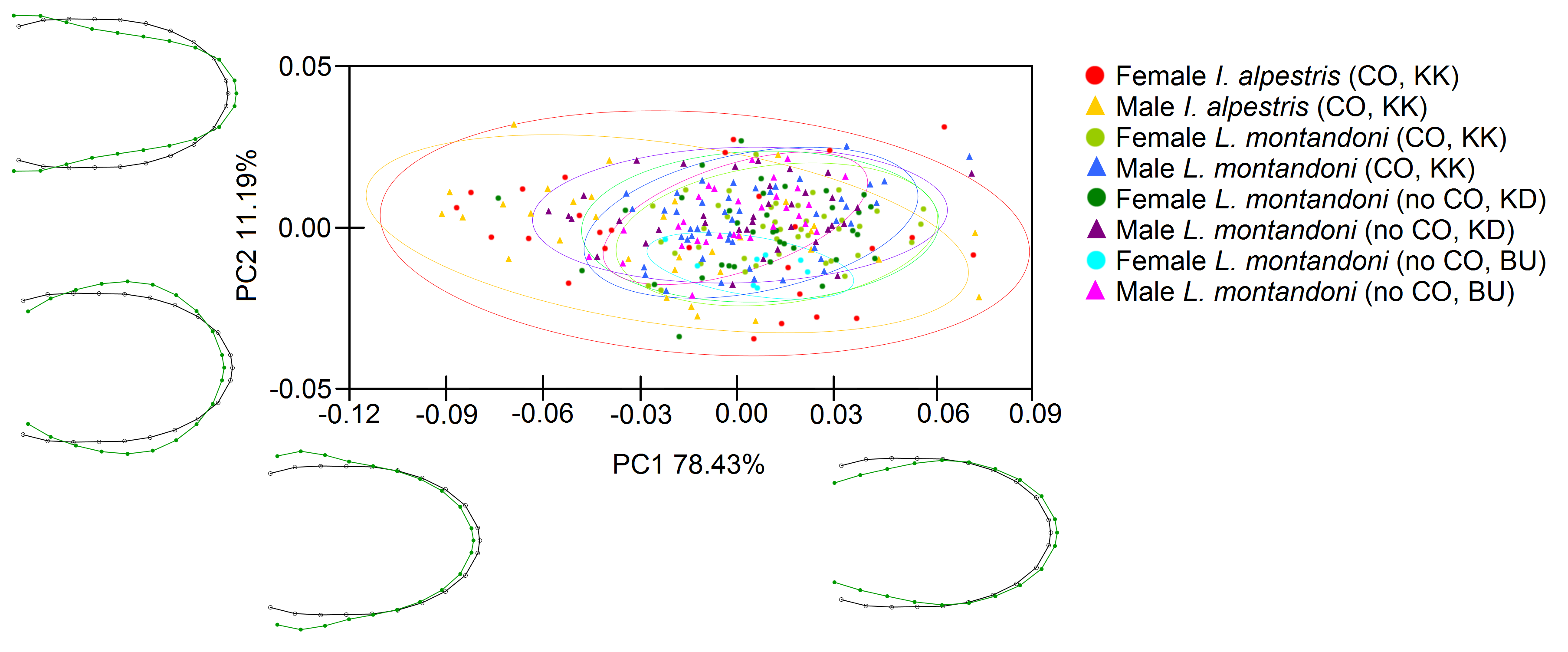

Supplement: Supplementary file 1 — Fig. 2S PCA of shapes of newt heads (ventral view). CO, co-occurrence with other newt species; no CO, absence of other newt species; black lines, mean shape; green lines, shape representing a specific CV; KK, a population from Kurov Kurovskie sedlo; KD, a population from Krize pod dedina, BU, a population from Bardejov urbamovka (TIFF 400 kb) [file 435_2017_366_MOESM1_ESM.tif]

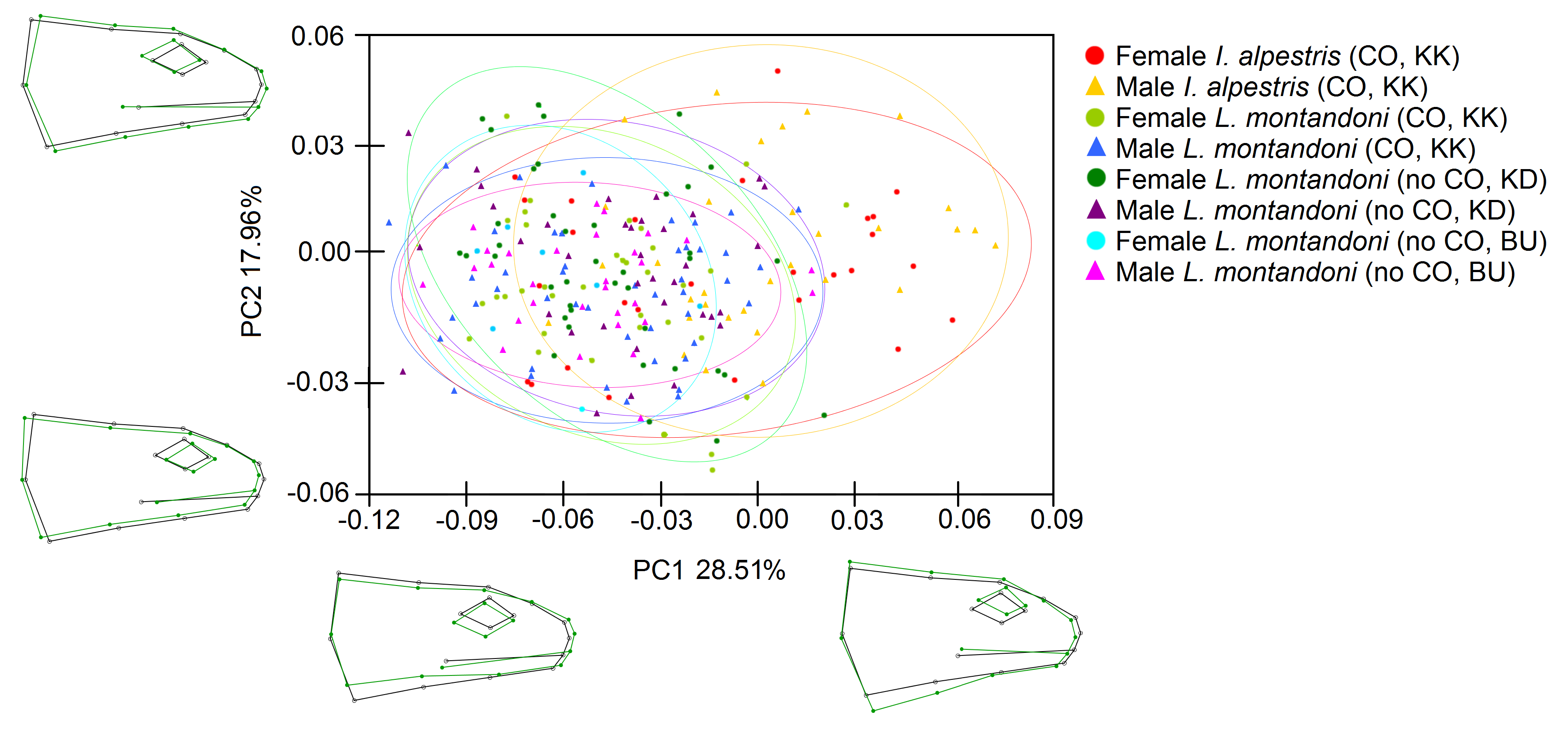

Supplement: Supplementary file 2 — Fig. 1S PCA of shapes of newt heads (lateral view). CO, co-occurrence with other newt species; no CO, absence of other newt species; black lines, mean shape; green lines, shape representing a specific CV; KK, a population from Kurov Kurovskie sedlo; KD, a population from Krize pod dedina; BU, a population from Bardejov urbamovka (TIFF 509 kb) [file 435_2017_366_MOESM2_ESM.tif]
