# Supplementary material for: Robustness of newt heads in condition of co-existence: a case of the Carpathian newt and the alpine newt
Source: Zoomorphology. 2017 Jul 19;136(4):511–21. doi: 10.1007/s00435-017-0366-7 (PMC5653732; doi:10.1007/s00435-017-0366-7)
Supplement: Supplementary file 3 — Supplementary material 3 (DOCX 13 kb) [file 435_2017_366_MOESM3_ESM.docx]

Table 1S Results of the Procrustes MANOVA for two *Lissotriton montandoni* populations

| Effect | Sum of squares | Mean squares | df | F | P | Pillay’s trace | P |
| --- | --- | --- | --- | --- | --- | --- | --- |
| *Lateral view* | | | | | | | |
| Site | 0*.*02171192 | 0*.*0007237305 | 30 | 1.64 | 0.0916 |  |  |
| Sex | 0*.*01658816 | 0*.*0005529387 | 30 | 1.25 | 0.2723 |  |  |
| Site×Side | 0*.*01326810 | 0*.*0004422701 | 30 | 1.84 | 0.0672 | 0.54 | 0.0999 |
| *Ventral view* | | | | | | | |
| Site | 0*.*00075771 | 0*.*0000398795 | 19 | 2.03 | 0.0547 |  |  |
| Side | 0*.*00035930 | 0*.*0000189106 | 19 | 1.20 | 0.3470 |  |  |
| Site×Side | 0*.*00029921 | 0*.*0000157478 | 19 | 0.32 | 0.9964 |  |  |
| Sex | 0*.*00372371 | 0*.*0000489962 | 76 | 1.03 | 0.0674 | 0.44 | 0.1233 |
