# Supplementary material for: Robustness of newt heads in condition of co-existence: a case of the Carpathian newt and the alpine newt
Source: Zoomorphology. 2017 Jul 19;136(4):511–21. doi: 10.1007/s00435-017-0366-7 (PMC5653732; doi:10.1007/s00435-017-0366-7)
Supplement: Supplementary file 4 — Supplementary material 4 (DOCX 13 kb) [file 435_2017_366_MOESM4_ESM.docx]

Table 1S. Descriptive statistics of centroid sizes

| Population | Female | | | Male | | | Female | | | Male | | |
| --- | --- | --- | --- | --- | --- | --- | --- | --- | --- | --- | --- | --- |
|  | Mean | SD | N | Mean | SD | N | Mean | SD | N | Mean | SD | N |
|  | *Ventral view* | | | | | | *Lateral view* | | | | | |
| IA | 23.07 | 1.43 | 28 | 21.95 | 1.51 | 33 | 13.37 | 0.69 | 28 | 14.07 | 0.57 | 33 |
| LM (CO) | 20.86 | 0.81 | 40 | 19.64 | 0.80 | 46 | 13.63 | 0.88 | 40 | 14.99 | 0.80 | 46 |
| LM (no CO, KD) | 21.06 | 1.04 | 36 | 20.38 | 0.86 | 37 | 14.34 | 0.63 | 36 | 15.14 | 1.39 | 37 |
| LM (no CO, BU) | 21.02 | 1.26 | 8 | 19.53 | 1.12 | 30 | 14.63 | 0.33 | 8 | 14.34 | 0.93 | 30 |

SD – standard deviation, N – number of individuals, LM – *Lissotriton montandoni*, IA - *Ichthyosaura alpestris,* CO – co-occurrence with other newt specie, no CO - absence of other newt specie, KD – a population from Krize pod dedina, BU – a population from Bardejov urbamovka.
